# Rapid Recovery Pathway for Postoperative Treatment of Adolescent Idiopathic Scoliosis

**DOI:** 10.5435/JAAOSGlobal-D-20-00220

**Published:** 2021-03-10

**Authors:** Eli S. Ahdoot, Juston Fan, Afshin Aminian

**Affiliations:** From the Orthopaedic Surgery, Riverside University Health System Medical Center, Moreno Valley, CA (Dr. Ahdoot, Dr. Fan), and the Orthopedic Institute, CHOC Children's, Orange, CA (Dr. Aminian).

## Abstract

Postoperative pain protocols play a critical role in recovery and prognosis. Rapid recovery pathway (RRP) is a novel multimodal postoperative analgesic platform with accelerated rehabilitation.

**Methods:**

A retrospective review of 44 patients with adolescent idiopathic scoliosis who underwent posterior spinal fusion between 2014 and 2016 was conducted. Outcomes of a conventional postoperative pain pathway were compared with patients who received RRP postoperatively.

**Results:**

RRP patients had shorter length of stay (3.3 vs 4.4 days, *P* < 0.0001), duration with Foley (1.4 vs 2.3 days, *P* = 0.01), and fewer days for physical therapy clearance (2.2 vs 3.5 days, *P* < 0.0001). Overall pain score for RRP patients was lower (1.6 vs 2.9, *P* = 0.0005). The number of days with patient-controlled analgesia was shorter (1.7 vs 2.6 days, *P* = 0.002), and daily pain scores were consistently lower in RRP. Overall narcotic use was not significantly different (*P* = 1).

**Conclusion:**

Implementation of a standardized RRP with multimodal pain management and early mobilization strategies resulted in reduced daily and overall pain scores, earlier clearance by physical therapy, decreased length of stay, and patient-controlled analgesia usage, but overall no difference in narcotic consumption.

**Level of evidence:**

Level III, Retrospective Cohort Study

Adolescent idiopathic scoliosis (AIS) is the most common type of scoliosis in children of age 10 to 18 years.^1^ There is an incidence of 3% for curves between 10° and 20°, and 0.3% for curves greater than 30°.^2^ AIS also shows a female predominance with a 10:1 female-to-male ratio. Spinal arthrodesis or posterior spinal fusion (PSF) has become the mainstay of treatment for severe AIS to correct and prevent further progression of the spinal deformity while preserving pulmonary function.^3^ Postoperative pain protocols play a critical role in quicker recovery and improved prognosis and are an important area of study and improvement.^4,5,6,7,8^ Rapid recovery pathway (RRP) is a novel multimodal analgesic platform with accelerated rehabilitation, which was implemented at our institution to better control pain with less narcotic use, ultimately leading to a quicker recovery.^9^ The risk of opioid-induced hyperalgesia, which refers to a phenomenon where opioid administration results in lowering of pain threshold, could also be reduced.^10,11^ Furthermore, a 22% decrease in postoperative hospital costs was found using an accelerated pathway.^12^ The goal of this study was to evaluate the outcomes of the RRP in comparison with the conventional pain pathway in adolescent patients who underwent PSF for AIS treated at our institution. We hypothesized that patients undergoing the RRP would have earlier hospital discharges, diminished pain, and less narcotic consumption.

## Methods

### Study Design, Setting, and Population

We have done a retrospective review of two cohorts of patients with the diagnosis of AIS who underwent PSF at our institution. Patients were broken up into two cohorts based on the postoperative treatment protocol received, either the conventional pain pathway or the RRP. Patients in the conventional pain pathway cohort had their surgeries done in the summer of 2014. Patients in the RRP had their surgeries done in the summer of 2016. All surgeries were done by a single surgeon without change in technique, technology, or implants. Inclusion criteria included all postoperative AIS patients treated with PSF. Exclusion criteria included those individuals with neuromuscular, congenital, or nonidiopathic scoliosis, anterior spinal fusions, or any intraoperative complication, including hemorrhage, coagulation, or loss of motor signal that required postoperative intensive care unit monitoring greater than 24 hours.

### Standard Protocol Approvals, Registrations, and Patient Consents

This study was done with the approval of the Children's Hospital of Orange County Institutional Review Board.

### Treatment Protocols

The conventional pain pathway had been used until RRP was implemented at our site in 2016. Patients in the conventional pain pathway had a postoperative pain protocol that included 24-hour monitoring in the pediatric intensive care unit, a 24-hour period of nothing by mouth starting after completion of the procedure, physical therapy (PT) on postoperative day 1, scheduled weight-based dosing of ibuprofen, and intravenous (IV) dilaudid through patient-controlled analgesia (PCA) with a gradual transition to oral pain medications. Patients in the RRP had a postoperative pain protocol that included a preoperative oral dose of gabapentin 600 mg, a full diet beginning on the day of surgery, 24 hours of postoperative IV acetaminophen, IV dilaudid through PCA, IV ketorolac for 48 hours beginning postoperative day 1, hydrocodone/acetaminophen 5/325 mg orally scheduled every 6 hours, oral gabapentin 300 mg three times a day for 7 days, oral diazepam 2 mg as needed three times per day, and PT to be initiated on the day of surgery. Dosing of diazepam was standardized for consistency and ease of administration instead of weight-based dosing. Other medications were administered on a weight-based scale if no dosage was specified.

### Statistical Analysis

The primary outcomes for our study were length of stay (LOS), time to Foley catheter removal determined by ambulation status, overall pain score measured by the visual analog score, postoperative hospital day that the patient was deemed safe for discharge by PT, overall narcotic requirement in mg/kg morphine equivalent calculated through the Opiate Equianalgesic Dosing Chart from the University of North Carolina Hospitals, number of doses of benadryl and ondansetron, and number of days with PCA.^13^ Patients' pain score and narcotic requirement were measured at each day before discharge. Thus, the differences of daily pain score and narcotic requirement were also examined as the secondary outcomes. The mean and SD were provided for all outcomes in the conventional pathway and RRP group. Multivariate linear regression was used to examine the difference in LOS, time to Foley catheter removal, the postoperative hospital day that the patient was deemed safe for discharge by PT, number of doses of benadryl and ondansetron, and number of days with PCA with adjustment for the number of spinal levels fused which reflects patients' disease severity. A mixed-effect model was conducted to compare overall pain score and narcotic requirement between groups with number of spinal levels fused and day of surgery as the fixed effect and a random effect to account for the within-subject correlation. For pain score and narcotic requirement, only the day of surgery and the next 3 days were used in the analysis for comparison because most patients were discharged from the hospital after day 4. A Bonferroni correlation was used to adjust for the multiple comparisons among primary outcomes.

## Results

**Figure 1:**
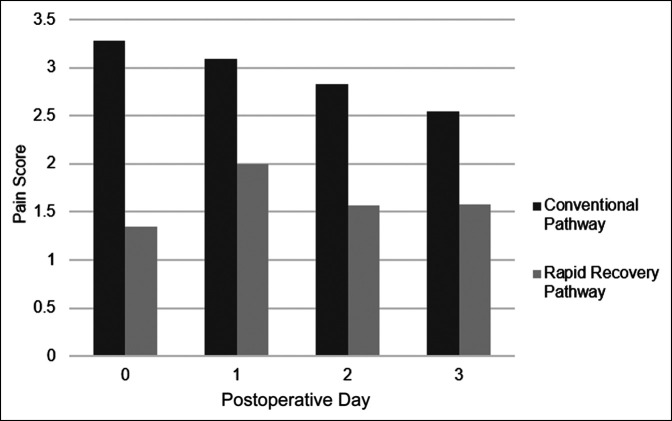
Bar chart showing postoperative pain comparison. Visual depiction of the level of pain for each postoperative day for the conventional pathway and RRP. RRP = rapid recovery pathway.

Of the 44 patients studied, 22 (50%) were in the conventional pathway and 22 were in the RRP who had undergone PSF for AIS for the same operative indications. Median age of patients at the time of surgery was 15 years for the conventional group with a range of 11 to 19 years and 16 years for the RRP group with a range of 11 to 21 years. In the RRP, there were 3 (14%) male patients and 19 (86%) female patients. In the conventional pathway group, there were 5 (23%) male patients and 17 (77%) female patients. Within the primary outcomes measurements, the mean (SD) LOS for the conventional pathway and the RRP were 4.41 (0.73) and 3.32 (0.57) days, respectively. Patients in the RRP group were discharged 1.08 days earlier than those in the conventional pathway group after adjusting for the number of spinal levels fused to address higher estimated blood loss and longer lengths of surgery with an increasing number of spinal levels fused (*P* < 0.0001; Table 1). Time to Foley catheter removal was significantly shorter in the RRP group with a difference of 0.85 days (*P* = 0.01). Time to clearance for safe discharge by PT was significantly shorter in the RRP. The mean (SD) number of hospital days before clearance for discharge by PT for the conventional pathway and the RRP were 3.50 (0.67) and 2.23 (0.53) days, respectively. RRP patients took 1.26 less days to be cleared for discharge by PT, adjusted for number of spinal levels fused (*P* < 0.0001; Table 1). Daily pain scores were less in the RRP than the conventional pathway. The overall pain score in RRP patients was 1.28 lower compared with the control group (*P* = 0.0005). The overall usage of narcotics was not significantly different between the two groups (*P* = 1.0000), but daily usage was less in the RRP group except for day of surgery, postoperative day 1, and postoperative day 2, although statistical significance was not reached (Table 2). The number of days with PCA for the RRP group was 0.89 days less than the conventional pain pathway group (*P* = 0.0024). Within our secondary outcome measurements, daily pain score was consistently lower in the RRP group compared with that of the conventional pathway group (Figure 1 and Table 2).

**Table 1:** Outcomes for Conventional Pathway Versus RRP

Factors	Conventional Pathway^a^	RRP^a^	Coefficient	Std	T value	Adjusted *P* Value
LOS, days	4.41 (0.73)	3.32 (0.57)	−1.08	0.2	−5.41	<0.0001
Hospital day Foley removed	2.32 (0.95)	1.45 (0.67)	−0.85	0.25	−3.41	0.0104
Pain score	2.93 (1.75)	1.62 (1.40)	−1.28	0.31	−4.13	0.0005
Hospital day cleared by PT	3.50 (0.67)	2.23 (0.53)	−1.26	0.18	−6.87	<0.0001
Narcotic requirement	14.91 (19.26)	25.31 (106.8)	12.22	11.29	1.08	1
No. doses of benadryl and ondansetron	4.50 (2.48)	3.09 (2.69)	−1.41	0.79	−1.79	0.5707
No. days with PCA	2.59 (0.96)	1.68 (0.48)	−0.89	0.23	−3.9	0.0024

LOS = length of stay, PCA = patient-controlled analgesia, PT = physical therapy, RRP = rapid recovery pathway

a Data are presented as mean (SD).

**Table 2:** Outcomes for Conventional Pathway Versus RRP

Factors	Day	Conventional Pathway		RRP		Coefficient	Std	T value	Adjusted *P* Value
		Count	Mean (SD)	Count	Mean (SD)				
Pain score	0	22	3.28 (2.40)	22	1.35 (1.36)	−1.9	0.6	−3.19	0.0028
	1	22	3.09 (1.50)	22	2.00 (1.58)	−1.07	0.47	−2.28	0.0281
	2	22	2.83 (1.12)	22	1.57 (1.42)	−1.21	0.38	−3.23	0.0024
	3	22	2.54 (1.74)	22	1.58 (1.22)	−0.95	0.46	−2.06	0.0454
	4	20	2.32 (2.97)	4	3.17 (2.42)				—-
	5	8	2.10 (0.97)	0	—				—-
	6	1	2.00 (−)	0	—				—
Narcotic Usage	0	21	38.98 (24.69)	22	74.21 (207.4)	44.03	42.7	1.03	0.3086
	1	22	4.56 (8.51)	22	11.90 (17.43)	7.83	4.08	1.92	0.0619
	2	22	5.65 (4.50)	22	8.49 (5.11)	2.83	1.47	1.92	0.0621
	3	22	11.54 (7.29)	21	5.76 (5.11)	−5.81	1.96	−2.97	0.0051
	4	18	11.47 (8.62)	7	4.42 (3.41)				—
	5	9	7.13 (3.56)	0	—				—
	6	2	3.33 (0.00)	0	—				—

PCA = patient-controlled analgesia, RRP = rapid recovery pathway

## Discussion

With the advent of PSF in patients with AIS, we have seen several advancements in postoperative recovery.^14^ However, further improvements in pain control, narcotic consumption, and rehabilitation are warranted to maximize patient-oriented outcomes, minimize procedure-related morbidity, and decrease costs in total perioperative care.^15^ It is well known that the immediate postoperative period may present with a number of innate challenges including pain control, delayed mobilization, and numerous opioid-related adverse effects such as nausea, vomiting, abdominal pain, ileus, and opioid-induced hyperalgesia.^4,10,11^ Pain control can be difficult in patients undergoing multiple level spinal fusions but more so in children and adolescents. Several postoperative protocols have been designed to diminish pain and enhance recovery in adolescent patients undergoing PSF with many involving a combination of IV and oral opioids, bed rest, and strict nothing by mouth. With the advent of multimodal analgesia, other surgical subspecialties, including total joint arthroplasty and colon surgery, have seen benefits in the perioperative period.^15^ The RRP was designed with a multidisciplinary team to accelerate rehabilitation by decreasing pain, opioid-related complications, and LOS. Ladha et al demonstrated that the United States had the highest average dose of postoperative opioid prescriptions compared with Canada and Sweden. Further scrutiny into prescribing practices of opioid pain medications is warranted.^16^ Although previous research has shown that RRP decreased LOS and hospital costs by 22%, our main goal was to report our success with the implementation of this new protocol and evaluate pain scores and narcotic consumption at our institution.^7,9,15^ We conducted a detailed, surgeon-specific comparison of patients treated with the conventional pain pathway with those treated with the newly implemented RRP. Our results indicated that the RRP group resulted in a decrease in LOS, decrease in time to PT clearance, earlier Foley catheter removal, and decrease in daily and overall pain scores. We did not find a difference in overall narcotic consumption between protocols. This may be explained by an earlier transition to oral, around the clock opioid administration. PCA was used on average of 0.89 days less compared with the conventional pain pathway. The amount of antiemetic medications used showed no statistically significant difference, although a lower trend was seen within the RRP group. It is our impression that those individuals with the multimodal analgesia protocol were more awake, alert, mobile, and comfortable as demonstrated by earlier times to PT clearance, Foley removal based on ambulation status, daily pain scores, and hospital discharge. Our transition to an accelerated protocol proved successful to our institution focusing on early ambulation, pain management, and standardized postoperative care.

This study is limited by its retrospective design and/or selection bias. Differences may be due to the hospital staff from preoperative nurses, anesthesia, postoperative, and floor nurses being aware of a newly implemented protocol. Another contributing factor may have been added emphasis on collecting pain scores by nursing and hospital administration in the RRP. In addition, a limitation of this study is that we do not have pain data after discharge. Another limitation is not having satisfaction surveys from parents and patients regarding their perception of the postoperative care. Although these would provide a baseline understanding of patient satisfaction, satisfaction surveys were not collected during the traditional protocol, and there would have been no means for comparison. Postoperative ileus and complications were not accounted for or compared from the conventional pathway with the RRP group. Return to the emergency department was also not documented for complications to be compared between groups. Despite these limitations, this study demonstrates that an accelerated protocol for PSF for the treatment of AIS can enhance recovery by earlier mobilization, earlier diet tolerance, and earlier hospital discharge.
